# Supplementary material for: A standardized method for plasma extracellular vesicle isolation and size distribution analysis
Source: PLoS One. 2023 Apr 28;18(4):e0284875. doi: 10.1371/journal.pone.0284875 (PMC10146456; doi:10.1371/journal.pone.0284875)
Supplement: S1 File — Step-by-step protocol, also available on protocols.io. (PDF) [file pone.0284875.s001.pdf]

Sep 21, 2022

Human Sample Processing and Isolation of Extracellular Vesicles with Size Exclusion Chromatography

J. Nathaniel Diehl[1], Amelia Ray[1], Lauren B. Collins[1], Andrew Peterson[2,3], Kyle C. Alexander[2,3], John S. Ikonomidis[2,3], Adam W. Akerman[2,3]

[1]University of North Carolina School of Medicine, Chapel Hill, North Carolina;

[2]Department of Surgery, University of North Carolina – Chapel Hill, Chapel Hill, North Carolina;

[3]Division of Cardiothoracic Surgery, University of North Carolina – Chapel Hill, Chapel Hill, North Carolina

1 Works for me Share

dx.doi.org/10.17504/protocols.io.3byl4jeb2lo5/v1

nate_diehl

ABSTRACT

This protocol details the steps necessary to isolate circulating plasma extracellular vesicles (EVs) from human peripheral blood samples. This protocol utilizes an automated fraction collector (AFC) and qEV size-exclusion chromatography columns from IZON Science. This is intended to serve as the first step in the workflow for EV quantification along with “Measurement of extracellular vesicles with tunable resistance pulse sensing (TRPS).”

ATTACHMENTS

ix3ebwxfp.docx

DOI

dx.doi.org/10.17504/protocols.io.3byl4jeb2lo5/v1

PROTOCOL CITATION

J. Nathaniel Diehl, Amelia Ray, Lauren B. Collins, Andrew Peterson, Kyle C. Alexander, John S. Ikonomidis, Adam W. Akerman 2022. Human Sample Processing and Isolation of Extracellular Vesicles with Size Exclusion Chromatography. **protocols.io** https://protocols.io/view/human-sample-processing-and-isolation-of-extracell-cft9tnr6

KEYWORDS

Extracellular vesicles, EVs, Exosomes, TRPS, Tunable resistive pulse sensing, Human plasma, Automatic fraction collector, AFC, qEV, Size exclusion chromatography, qNano Gold

Citation: J. Nathaniel Diehl, Amelia Ray, Lauren B. Collins, Andrew Peterson, Kyle C. Alexander, John S. Ikonomidis, Adam W. Akerman Human Sample Processing and Isolation of Extracellular Vesicles with Size Exclusion Chromatography https://dx.doi.org/10.17504/protocols.io.3byl4jeb2lo5/v1

This is an open access protocol distributed under the terms of the **Creative Commons Attribution License**

LICENSE

This is an open access protocol distributed under the terms of the Creative Commons Attribution License, which permits unrestricted use, distribution, and reproduction in any medium, provided the original author and source are credited

CREATED

Aug 26, 2022

LAST MODIFIED

Sep 21, 2022

OWNERSHIP HISTORY

Aug 26, 2022 madhavi.d

Sep 06, 2022 nate_diehl

PROTOCOL INTEGER ID

69217

Citation: J. Nathaniel Diehl, Amelia Ray, Lauren B. Collins, Andrew Peterson, Kyle C. Alexander, John S. Ikonomidis, Adam W. Akerman Human Sample Processing and Isolation of Extracellular Vesicles with Size Exclusion Chromatography https://dx.doi.org/10.17504/protocols.io.3byl4jeb2lo5/v1

This is an open access protocol distributed under the terms of the **Creative Commons Attribution License**

GUIDELINES

- All human blood samples should be handled according to the Centers for Disease Control (CDC) universal blood and body fluid collection guidelines. Additionally, sample handling and processing should follow the Occupational Safety and Health Administration (OSHA) blood borne pathogens procedures to prevent possible pathogen transmission.
- This protocol details the extraction of EVs from human peripheral blood samples. All human subjects research should be reviewed and approved by the site-specific Institutional Review Board (IRB) prior to proceeding.
- qEVsingle columns are used in this protocol. Newer generation multi-use columns can also be used if column-specific instructions are followed.

Citations:

1. IZON AFC quick start guide ◊
   https://files.izon.com/hubfs/Manuals,%20Technical%20Notes%20and%20Customer%20Support/AFC/AFC%20Quick%20Start%20Guide.pdf
2. IZON Full Manual ◊
   https://f.hubspotusercontent30.net/hubfs/4136435/Manuals,%20Technical%20Notes%20and%20Customer%20Support/AFC/rotational-afc-user-manual-RA1-OQ-001.pdf

Additional Notes:

- If the AFC continuously stops running in the middle of an extraction and it is not due to running out of buffer, check that the tubing is not obstructed by removing it from the tubing apparatus and confirming that liquid can be pushed through. Tubing can be replaced if necessary.
- For further troubleshooting or information, please refer to the IZON AFC Quick Start Guide and the IZON AFC Full Manual.

Citation: J. Nathaniel Diehl, Amelia Ray, Lauren B. Collins, Andrew Peterson, Kyle C. Alexander, John S. Ikonomidis, Adam W. Akerman Human Sample Processing and Isolation of Extracellular Vesicles with Size Exclusion Chromatography https://dx.doi.org/10.17504/protocols.io.3byl4jeb2lo5/v1

This is an open access protocol distributed under the terms of the **Creative Commons Attribution License**

MATERIALS TEXT

- Sterile alcohol prep pads Fisher **Scientific Catalog #22-363-750**
- 25-gauge Safety-Lok needle and collection set **Bd Catalog #367285**
- BD Vacutainer EDTA tube **Fisher Scientific Catalog #23-021-013**
- Cryogenic tubes with screw cap **Fisher Scientific Catalog #03-337-7Y**
- Microcentrifuge tubes **Eppendorf Catalog #022364111**
- qEVsingle **35 nm** Legacy column, IZON, PC#SP6
- 200 µL filtered pipet tips **Millipore Sigma Catalog #CLS4823-960EA**
- 1000 µL filtered pipet tips **Millipore Sigma Catalog #CLS4809-1000EA**
- **10 mL** Sterile syringe with Luer lock, Fisher Scientific, Cat#14-955-460
- 0.22 µm Luer lock inlet filters **Thomas Scientific Catalog #1176G49**
- PBS Tablets **Thermo Fisher Catalog #18912014**
- Filtered (**0.22 µm**) deionized water

Equipment:

- Ultra-low temperature (**-80 °C**) freezer
- Standard manual defrost laboratory (**-20 °C**) freezer
- Refrigerated microcentrifuge **Eppendorf Catalog #022620700**
- Clinical Centrifuge, Globe Scientific, Item# GCC-E
- qEV Automatic fraction collector (AFC)
- **20 µL** - **200 µL** pipette
- **100 µL** - **1000 µL** pipette

SAFETY WARNINGS

Appropriate personal protective equipment (PPE) should be worn (nitrile gloves, safety goggles, and lab coat). Store all organic solvents in a flammable storage cabinet in accordance with institution policy. Utilize biohazard waste containers for sample

Citation: J. Nathaniel Diehl, Amelia Ray, Lauren B. Collins, Andrew Peterson, Kyle C. Alexander, John S. Ikonomidis, Adam W. Akerman Human Sample Processing and Isolation of Extracellular Vesicles with Size Exclusion Chromatography https://dx.doi.org/10.17504/protocols.io.3byl4jeb2lo5/v1

This is an open access protocol distributed under the terms of the **Creative Commons Attribution License**

waste.

BEFORE STARTING

- Ensure that you have an adequate quantity of buffer (1x sterile-filtered PBS)
- If you already have frozen human plasma samples, skip to “EV Isolation”.

Human plasma collection

1 Collect **5 mL** peripheral venous blood by a trained nurse or phlebotomist into prelabeled EDTA-coated evacuated tubes.

EDTA anticoagulant is recommended for isolation of EVs.

2 — 2h

Immediately after the sample is collected, mix the tube thoroughly and store at **Room temperature** (< **02:00:00**).

3 — 15m

Centrifuge whole blood at **2500 x g** for **00:15:00** at **Room temperature** .

4

Collect the topmost layer (plasma) of supernatant into a 15 mL conical tube.

4.1 Carefully avoid disruption of the next layer (buffy coat). Leave **1 cm** of plasma above the buffy coat.

5 — 15m

Centrifuge the plasma again at **2500 x g** for **00:15:00** at **Room temperature** .

6

Avoiding the bottom ~ **100 µL** of plasma, collect the topmost plasma into a new **15 mL** conical tube.

Citation: J. Nathaniel Diehl, Amelia Ray, Lauren B. Collins, Andrew Peterson, Kyle C. Alexander, John S. Ikonomidis, Adam W. Akerman Human Sample Processing and Isolation of Extracellular Vesicles with Size Exclusion Chromatography https://dx.doi.org/10.17504/protocols.io.3byl4jeb2lo5/v1

This is an open access protocol distributed under the terms of the **Creative Commons Attribution License**

7 Aliquot desired volumes into labeled freezer-safe microcentrifuge tubes.

We recommend a minimum aliquot volume of **250 µL** .

8 Snap freeze plasma fractions and store at **-81 °C** .

EV Isolation 15m

9 15m

Thaw human plasma at **Room temperature** and centrifuge plasma **2500 x g** for **00:15:00** at **4 °C** .

10 Power on IZON automated fraction collector (AFC).

11 Select SETUP > calibration.

11.1 Follow the on-screen prompts provided by the AFC to calibrate the machine using the provided **10 g** weight.

12 Insert a new **35 nm** qEVsingle column into the column mount and allow the machine to register it.

The display will indicate specific column features once it has registered.

12.1 Column settings: qEV = **35 nm** , count = 4, size = **0.2 mL** , void = **0.8 mL** , sample = **0.15 mL** .

Citation: J. Nathaniel Diehl, Amelia Ray, Lauren B. Collins, Andrew Peterson, Kyle C. Alexander, John S. Ikonomidis, Adam W. Akerman Human Sample Processing and Isolation of Extracellular Vesicles with Size Exclusion Chromatography
https://dx.doi.org/10.17504/protocols.io.3byl4jeb2lo5/v1

This is an open access protocol distributed under the terms of the **Creative Commons Attribution License**

13 Follow on-screen prompts, click OK to proceed to next step.

13.1 Permit existing buffer + **2 mL** additional 1x sterile filtered PBS to flush the column.

14 Once flush has completed, click OK to proceed to next step.

14.1

Load **150 µL** human plasma sample in a drop-wise manner into the center of the column.

15 Click OK to proceed to next step.

AFC should move from flush position to collect column buffer into the central well.

16

Once the sample has completely entered the column, carefully load **2 mL** 1x PBS into the top of the column in a drop-wise manner.

16.1 Continue to add buffer as needed until the extraction is complete.

16.2 If the collection stops at this point, ensure adequate buffer remains above the beads in the column and click OK to proceed.

17 Once the AFC has finished the extraction, combine the first three fractions and discard or store the fourth.

The fourth fraction contains primarily protein and a very low concentration of EVs.

Citation: J. Nathaniel Diehl, Amelia Ray, Lauren B. Collins, Andrew Peterson, Kyle C. Alexander, John S. Ikonomidis, Adam W. Akerman Human Sample Processing and Isolation of Extracellular Vesicles with Size Exclusion Chromatography https://dx.doi.org/10.17504/protocols.io.3byl4jeb2lo5/v1

This is an open access protocol distributed under the terms of the **Creative Commons Attribution License**

18 Discard the used qEVsingle column and clean the central well with a kimwipe delicate task wipe.

> Ensure the central well is completely dry before next use.

19 Repeat steps 12 -18 for additional samples.

20 Remove peristaltic pump tubing from below the column mount by removing the plastic cover on the peristaltic pump.

21 Flush plastic tubing with **5 mL** filtered water using a 10 mL syringe and replace plastic tubing on AFC.

> This will prevent buildup of salt crystals in the tubing between uses.

22 Combine fractions 1-3 and immediately process for use on TRPS, store short term (1-2 weeks) at **-20 °C** , or store long term (> 2 weeks) at **-81 °C** .

Citation: J. Nathaniel Diehl, Amelia Ray, Lauren B. Collins, Andrew Peterson, Kyle C. Alexander, John S. Ikonomidis, Adam W. Akerman Human Sample Processing and Isolation of Extracellular Vesicles with Size Exclusion Chromatography https://dx.doi.org/10.17504/protocols.io.3byl4jeb2lo5/v1

This is an open access protocol distributed under the terms of the **Creative Commons Attribution License**
