# Supplementary material for: A standardized method for plasma extracellular vesicle isolation and size distribution analysis
Source: PLoS One. 2023 Apr 28;18(4):e0284875. doi: 10.1371/journal.pone.0284875 (PMC10146456; doi:10.1371/journal.pone.0284875)
Supplement: S2 File — Step-by-step protocol, also available on protocols.io. (PDF) [file pone.0284875.s002.pdf]

Sep 22, 2022

Measurement of Extracellular Vesicles with Tunable Resistive Pulse Sensing

J. Nathaniel Diehl[1], Amelia Ray[1], Lauren B. Collins[1], Andrew Peterson[2,3], Kyle C. Alexander[2,3], John S. Ikonomidis[2,3], Adam W. Akerman[2,3]

[1]University of North Carolina School of Medicine, Chapel Hill, North Carolina;

[2]Department of Surgery, University of North Carolina – Chapel Hill, Chapel Hill, North Carolina;

[3]Division of Cardiothoracic Surgery, University of North Carolina – Chapel Hill, Chapel Hill, North Carolina.

1 Works for me Share

dx.doi.org/10.17504/protocols.io.ewov1ojnolr2/v1

nate_diehl

ABSTRACT

This protocol details the steps necessary to quantify the concentration and size distribution of plasma-derived extracellular vesicles (EVs). This protocol utilizes a qNano Gold Tunable Resistive Pulsing Sensing (TRPS) measurement system from IZON Science. This is intended to serve as the second step in the workflow for EV quantification along with “Human Sample Processing and Isolation of Extracellular Vesicles with Size Exclusion Chromatography.”

ATTACHMENTS

ix3fbwxfp.docx

DOI

dx.doi.org/10.17504/protocols.io.ewov1ojnolr2/v1

PROTOCOL CITATION

J. Nathaniel Diehl, Amelia Ray, Lauren B. Collins, Andrew Peterson, Kyle C. Alexander, John S. Ikonomidis, Adam W. Akerman 2022. Measurement of Extracellular Vesicles with Tunable Resistive Pulse Sensing. **protocols.io** https://protocols.io/view/measurement-of-extracellular-vesicles-with-tunable-cfudtns6

KEYWORDS

Extracellular vesicles, EVs, Exosomes, TRPS, Tunable resistive pulse sensing, Human plasma, Automatic fraction collector, AFC, qEV, Size exclusion chromatography, qNano Gold, Plasma

Citation: J. Nathaniel Diehl, Amelia Ray, Lauren B. Collins, Andrew Peterson, Kyle C. Alexander, John S. Ikonomidis, Adam W. Akerman Measurement of Extracellular Vesicles with Tunable Resistive Pulse Sensing https://dx.doi.org/10.17504/protocols.io.ewov1ojnolr2/v1

This is an open access protocol distributed under the terms of the **Creative Commons Attribution License**

LICENSE

This is an open access protocol distributed under the terms of the Creative Commons Attribution License, which permits unrestricted use, distribution, and reproduction in any medium, provided the original author and source are credited

CREATED

Aug 26, 2022

LAST MODIFIED

Sep 22, 2022

OWNERSHIP HISTORY

Aug 26, 2022 madhavi.d

Sep 06, 2022 nate_diehl

PROTOCOL INTEGER ID

69221

GUIDELINES

- All human sample handling and processing should follow the Occupational Safety and Health Administration (OSHA) blood borne pathogens procedures to prevent possible pathogen transmission.
- This protocol details the measurement of EVs from plasma samples. All human subjects research should be reviewed and approved by the site-specific Institutional Review Board (IRB) prior to proceeding.
- This protocol uses the IZON qNano Gold TRPS instrument. If other TRPS measurement systems are used (e.g. IZON Exoid), this workflow will likely need to be adjusted slightly to incorporate alternate equipment.

Citations:

1. IZON qNano User Manual ◊ https://f.hubspotusercontent30.net/hubfs/4136435/Manuals,%20Technical%20Notes%20and%20Customer%20Support/qNano/qnano-user-manual-QN1-OQ-014.pdf.
2. IZON qNano Control Suite Software ◊ https://support.izon.com/qnano-control-suite-software.

Additional Notes:

1. 2x PBS is preferred for measuring exosomes (30-150 nm) because it decreases the background noise.
2. Strategies for nanopore opening: These troubleshooting strategies can be used in any combination to facilitate the stabilization or opening of a nanopore. These

Citation: J. Nathaniel Diehl, Amelia Ray, Lauren B. Collins, Andrew Peterson, Kyle C. Alexander, John S. Ikonomidis, Adam W. Akerman Measurement of Extracellular Vesicles with Tunable Resistive Pulse Sensing https://dx.doi.org/10.17504/protocols.io.ewov1ojnolr2/v1

This is an open access protocol distributed under the terms of the **Creative Commons Attribution License**

methods can also be used to help unclog a nanopore when a run is paused.

- **Tapping** → With the VPM nozzle in place, use your index finger to firmly tap the shielding cap 3-5 times. This can disrupt bubbles and assist with pore opening.

- **Clicking** → Remove pressure from system and open pressure valve. Remove VPM nozzle. Raise the shielding and set it on the nanopore such that the posts of the shielding cap are resting on the arms of the nanopore. Apply downward pressure and begin to rotate until the shielding cap abruptly "clicks" downward into place.

- **Pressure application device (PAD) use** → Remove pressure from system and open pressure valve. Remove VPM nozzle and the shielding cap. Center PAD over upper fluid cell and give 10 plunges. Repeat as necessary to unclog nanopore or reduce RMS noise.

- **Cleaning upper fluid cell** → Remove pressure from system and open pressure valve. Remove VPM nozzle. Remove shielding cap and upper fluid cell. Clean upper fluid cell with filtered water and dry completely. Gently wipe top of nanopore with edge of Kimwipe. Replace upper fluid cell and shielding cap. Carefully pipette 2x ME or sample into upper fluid cell, avoiding introduction of bubbles. Proceed with run.

- **Nanopore cleaning** → Remove pressure from system and open pressure valve. Remove VPM nozzle. Remove shielding cap and upper fluid cell. Clean upper fluid cell with filtered water and dry completely. Reduce stretch on nanopore to 42 mm and remove from posts. Rinse each side of nanopore with 500 µL 70% ethanol followed by 500 µL filtered water. Carefully dry each side of nanopore with edge of Kimwipe. Dry lower fluid cell with Kimwipe. Replace nanopore on the posts and rotate knob clockwise to achieve desired stretch. Pipet 75 µL 2x ME carefully into the lower fluid cell, avoiding the introduction of bubbles. Replace upper fluid cell and shielding cap. Carefully pipette 35 µL 2x ME into upper fluid cell and click "Turn On". Monitor current and RMS noise, proceed with other troubleshooting strategies as necessary.

- **Adjusting pore stretch** → once a pore has been used, it is possible that it will need to be stretched >47 mm. Test nanopore stability at increased stretch values (maximum recommended stretch is 48 mm).

Citation: J. Nathaniel Diehl, Amelia Ray, Lauren B. Collins, Andrew Peterson, Kyle C. Alexander, John S. Ikonomidis, Adam W. Akerman Measurement of Extracellular Vesicles with Tunable Resistive Pulse Sensing
https://dx.doi.org/10.17504/protocols.io.ewov1ojnolr2/v1

This is an open access protocol distributed under the terms of the **Creative Commons Attribution License**

MATERIALS TEXT

- Nanopore, IZON, NP100
- Calibration particles, IZON, CPC100 (**100 nm**)
- Filtered (**0.22 µm**) 70% Ethanol in water
- Filtered (**0.22 µm**) deionized water
- Microcentrifuge tubes (1.7 mL), VWR, Catalog #87003-296
- Kimtech Kimwipe delicate task wipes **Kimberly-Clark Catalog #34155**
- Tissue culture dish (10 cm) **Millipore Sigma Catalog #Z707651**
- 200 µL filtered pipet tips **Millipore Sigma Catalog #CLS4823-960EA**
- 1000 µL filtered pipet tips, VWR Universal Pipet Tips, Catalog #732-3639
- **10 mL** Sterile syringe with Luer lock, Henke-ject, Catalog #5100-x00v0
- 0.22 µm Luer lock inlet filters, VWR Syringe Filter, Catalog #514-1263
- PBS tablets, IZON, PC#RK3

Equipment:

- Standard manual defrost laboratory **-20 °C**) freezer
- Standard laboratory (**4 °C**) refrigerator
- Refrigerated microcentrifuge **Eppendorf Catalog #022620700**
- qNano Gold TRPS Measurement System
- **20 µL** - **200 µL** pipette
- **100 µL** - **1000 µL** pipette
- Digital calipers
- Computer with Windows 10 operating system with Izon Control Suite v3.4 installed.

SAFETY WARNINGS

Appropriate personal protective equipment (PPE) should be worn (nitrile gloves, safety goggles, and lab coat). Store all organic solvents in a flammable storage cabinet in accordance with institution policy. Utilize biohazard waste containers for sample waste.

BEFORE STARTING

- Ensure IZON software has been installed on computer that will be used to run qNano instrument.
- Reagents should be room temperature when used on the qNano. Allow adequate acclimation to ambient temperature prior to stretching the nanopore or beginning the experiment.

Citation: J. Nathaniel Diehl, Amelia Ray, Lauren B. Collins, Andrew Peterson, Kyle C. Alexander, John S. Ikonomidis, Adam W. Akerman Measurement of Extracellular Vesicles with Tunable Resistive Pulse Sensing https://dx.doi.org/10.17504/protocols.io.ewov1ojnolr2/v1

This is an open access protocol distributed under the terms of the **Creative Commons Attribution License**

Reagent and sample preparation

1 Prepare 2x phosphate-buffered saline (2x PBS).

> This can be stored at 4 °C 1-2 weeks.

1.1 Completely dissolve one PBS tablet in 100 mL deionized water.

2 Prepare and filter (0.22 µm) 2x Measuring Electrolyte (2x ME) daily.

2.1

Dilute 30 µL Wetting Concentrate in 10 mL 2x PBS. Filter (0.22 µm) prior to use.

3 Dilute calibration particles (CPC100) 1:1500 in 2x ME. Store at 4 °C 1-2 weeks.

4 10m

Thaw sample(s) at Room temperature and centrifuge 10000 x g for 00:10:00 .

> We recommend preparing and measuring two samples simultaneously given our experience with sample run time and nanopore stability.

5 10m

Dilute samples 1:10 in 2x ME and centrifuge 10000 x g for 00:10:00 .

- If concentration is unknown, begin with serial dilutions (1:5, 1:10, 1:20) with a target

Citation: J. Nathaniel Diehl, Amelia Ray, Lauren B. Collins, Andrew Peterson, Kyle C. Alexander, John S. Ikonomidis, Adam W. Akerman Measurement of Extracellular Vesicles with Tunable Resistive Pulse Sensing https://dx.doi.org/10.17504/protocols.io.ewov1ojnolr2/v1

This is an open access protocol distributed under the terms of the **Creative Commons Attribution License**

volume of 200 µL .

- Centrifugation helps prevent aggregation and clogging of the nanopore with larger debris.
- Diluted sample can be stored at -20 °C for 1-2 weeks.

Preparing and Wetting the nanopore

6

Plug-in qNano instrument and open the Izon Control Suite. Select “Classic capture.”

7 Remove the shielding cap and upper fluid cell and set aside on kimwipe.

8

Wet each side of the nanopore (NP100) with 500 µL 70% ethanol.

> We utilized a 10 cm tissue culture dish to keep nanopore off benchtop and to collect cleaning fluids.

9

Rinse nanopore with 500 µL filtered water to remove ethanol. Pat dry with kimwipe, careful not to apply pressure to the central nanopore.

10 1m

Wet the bottom electrode with 75 µL 70% ethanol, allow it to sit for 00:01:00 and then dry completely with kimwipe.

11

Rinse bottom electrode with 75 µL filtered water and dry completely with kimwipe. Proceed immediately to next step.

Citation: J. Nathaniel Diehl, Amelia Ray, Lauren B. Collins, Andrew Peterson, Kyle C. Alexander, John S. Ikonomidis, Adam W. Akerman Measurement of Extracellular Vesicles with Tunable Resistive Pulse Sensing https://dx.doi.org/10.17504/protocols.io.ewov1ojnolr2/v1

This is an open access protocol distributed under the terms of the **Creative Commons Attribution License**

12 Fit nanopore onto the posts with the serial number facing upwards and the long arm towards the user.

13 Stretch the nanopore by turning the knob clockwise and measure the stretch using the digital calipers. For initial measurement, stretch to **47 mm** and enter this value in the software interface.

13.1 Measure distance between three-prong posts opposite one another.

13.2 Once the initial stretch is confirmed and recorded, the software will track additional adjustments.

13.3 Begin recording the duration of time that the nanopore has been stretched. Nanopores typically begin to destabilize or overstretch when used >6-7 hours.

14

To wet the nanopore, slowly pipet **75 µL** 2x ME into the lower fluid cell being careful not to introduce bubbles.

- The lower fluid cell is the space between the bottom electrode and the bottom surface of the nanopore.
- Bubbles will contribute to significant background noise during measurement.

15

Clean the upper fluid cell with **500 µL** filtered water and dry completely by tapping firmly on a kimwipe or drying directly with a kimwipe.

16

Fit the upper fluid cell on the qNano above the nanopore and add **35 µL** 2x ME into the central well of the upper fluid cell, being careful to avoid introducing bubbles.

Citation: J. Nathaniel Diehl, Amelia Ray, Lauren B. Collins, Andrew Peterson, Kyle C. Alexander, John S. Ikonomidis, Adam W. Akerman Measurement of Extracellular Vesicles with Tunable Resistive Pulse Sensing https://dx.doi.org/10.17504/protocols.io.ewov1ojnolr2/v1

This is an open access protocol distributed under the terms of the **Creative Commons Attribution License**

Tips for pipetting: Approach the edge of the nanopore surface at a slight angle through the upper fluid cell. Be careful not to disturb the nanopore surface, and slowly pipette **35 µL** fluid. Do not push beyond the first stop on the pipet as this will introduce air/bubbles into the fluid.

17 Place shielding cap over the upper fluid cell and fit the Variable Pressure Module (VPM) nozzle into the upper fluid cell.

18 Set voltage to 0.10 V and select "Turn On". Apply 20 mbar pressure by closing the pressure valve and adjusting the pressure bar. Allow to run for **00:04:00** - **00:05:00** for pore opening/wetting. 9m

19 Ensure that the nanopore is open completely. See "Additional notes ->Strategies for pore opening" for strategies to promote pore opening and stabilization.

- It is unlikely that the pore will open completely without tapping/clicking.
- An expected stable baseline current at 0.10 V is 10-38 nA.

20 Close the pressure valve and increase the pressure with the pressure bar to 8 mbar-10 mbar.

21 Slowly increase the voltage in a step-wise manner until a current of 110-135 nA is reached.

- We recommend targeting 120 nA for a working current.
- The RMS noise should be <10. If it is above 10, refer to "Strategies for pore opening".
- The process of opening and stabilizing the nanopore can take **00:15:00** - **01:00:00** .

Recording calibration particles

22 Once the current is stable at ~120 nA and the RMS noise is <10, remove the shielding cap and upper fluid cell.

Citation: J. Nathaniel Diehl, Amelia Ray, Lauren B. Collins, Andrew Peterson, Kyle C. Alexander, John S. Ikonomidis, Adam W. Akerman Measurement of Extracellular Vesicles with Tunable Resistive Pulse Sensing https://dx.doi.org/10.17504/protocols.io.ewov1ojnolr2/v1

This is an open access protocol distributed under the terms of the **Creative Commons Attribution License**

23 Clean upper fluid cell by tapping firmly on kimwipe and/or drying with kimwipe directly.

24 Gently use the edge of a kimwipe to dry the top of the nanopore.

25 Gently use the edge of a kimwipe to dry the top of the nanopore.

> **Tips for wicking:** Roll the kimwipe to a fine point to allow greater precision in liquid removal.

26

Replace upper fluid cell and shielding cap, add **35 µL** of diluted calibration particles (careful not to introduce bubbles during pipetting).

> **Tips for pipetting:** Approach the edge of the nanopore surface at a slight angle through the upper fluid cell. Be careful not to disturb the nanopore surface, and slowly pipette **35 µL** fluid. Do not push beyond the first stop on the pipet as this will introduce air/bubbles into the fluid.

27 Replace VPM nozzle, close pressure valve, and set pressure bar to 3 mbar ('Working Pressure 1').

- The actual pressure reading in the software will be slightly different from the pressure set with the pressure bar.
- It is recommended to start at lower pressures for smaller pores, such as the NP100.

28 Observe particle size and adjust pore stretch and voltage accordingly.

- If current is higher/lower than 120 nA, adjust voltage such that the current is 116-124 nA.

Citation: J. Nathaniel Diehl, Amelia Ray, Lauren B. Collins, Andrew Peterson, Kyle C. Alexander, John S. Ikonomidis, Adam W. Akerman Measurement of Extracellular Vesicles with Tunable Resistive Pulse Sensing https://dx.doi.org/10.17504/protocols.io.ewov1ojnolr2/v1

This is an open access protocol distributed under the terms of the **Creative Commons Attribution License**

- If the particles appearing in the interface are outside of 0.2-0.25 % relative particle size, you can adjust the stretch on the pore to target this range. The voltage may need to be adjusted to keep the current in range.

29 Note the voltage, stretch, RMS noise, and current. These should be kept constant for all calibration and sample readings.

30

Start recording calibration particles at 'Working Pressure 1' (e.g. 3 mbar). Permit recording of a minimum of 500 particles before stopping the recording.

> If the RMS noise suddenly increases, it is likely that the nanopore has clogged. Pause the run and tap the shielding cap firmly. Unpause run to continue recording if the RMS noise <10. If this does not reduce RMS noise, you can refer to "Strategies for pore opening" for additional strategies.

31 Remove pressure by pulling out pressure bar completely and then label the sample. An example label is included below:

- Investigation: Experiment name and date.
- Nanopore ID: serial number.
- Nanopore Part#: NP100.
- Sample ID: calibration#1_pressure3_dilution.1.1500.
- Raw concentration (calibration only): 1.7e13.
- Dilution: 1500.
- Pressure: 3 (the software resets this value, include pressure setting in Sample ID).
- Electrolyte ID: 2x ME.

32 Set the pressure bar to 'Working Pressure 2' (6 mbar recommended). Repeat steps 29-30.

33 Set the pressure bar to 'Working Pressure 3' (9 mbar recommended). Repeat steps 29-30.

> Recording calibration particles at three working pressures allows flexibility in recording samples at equivalent working pressures.

Citation: J. Nathaniel Diehl, Amelia Ray, Lauren B. Collins, Andrew Peterson, Kyle C. Alexander, John S. Ikonomidis, Adam W. Akerman Measurement of Extracellular Vesicles with Tunable Resistive Pulse Sensing https://dx.doi.org/10.17504/protocols.io.ewov1ojnolr2/v1

This is an open access protocol distributed under the terms of the **Creative Commons Attribution License**

Sample/calibration particle changeover

34 Remove the shielding cap and upper fluid cell.

35

Clean upper fluid cell by pipetting 500 µL filtered water into upper fluid cell and then tapping firmly on kimwipe and/or drying with kimwipe directly.

36 Gently use the edge of a kimwipe to dry the top of the nanopore.

37 Replace upper fluid cell and shielding cap.

38

Add 35 µL of 2x ME to upper fluid cell (careful not to introduce bubbles during pipetting).

39 Replace VPM nozzle, close pressure valve, and set pressure bar to 20 mbar (max pressure).

20s

39.1 If the rate is below 15 particles/min after 00:00:10 , proceed with the next sample/calibration. If the rate is above 15 particles/min after 00:00:10 , repeat steps 33-38.

40 If particle rate is <15 particles/min, remove shielding cap and upper fluid cell.

41 Tap dry upper fluid cell and gently use a kimwipe to clean top of nanopore.

42 Replace upper fluid cell and shielding cap.

Citation: J. Nathaniel Diehl, Amelia Ray, Lauren B. Collins, Andrew Peterson, Kyle C. Alexander, John S. Ikonomidis, Adam W. Akerman Measurement of Extracellular Vesicles with Tunable Resistive Pulse Sensing https://dx.doi.org/10.17504/protocols.io.ewov1ojnolr2/v1

This is an open access protocol distributed under the terms of the **Creative Commons Attribution License**

Sample recording

43 Follow sample changeover protocol as above when switching from calibration particles to samples or between samples.

> This step ensures accurate measurements and avoids mixing or diluting samples.

44

Add 35 µL dilute sample to upper fluid cell. Replace the VPM nozzle, close the pressure valve, and set pressure bar to desired working pressure.

> Tips for pipetting: approach the edge of the nanopore surface at a slight angle through the upper fluid cell. Be careful not to disturb the nanopore surface, and slowly pipette 35 µL fluid. Do not push beyond the first stop on the pipet as this will introduce air/bubbles into the fluid.

45 Ensure that stretch and voltage settings are the same for as those used for the corresponding calibration particles. Additionally, ensure the current is within the 116-124 nA range and the RMS noise is <10.

46

Start recording sample at ‘Working Pressure 1’ (e.g. 3 mbar). Permit recording of a minimum of 500 particles before stopping the recording.

> - If the RMS noise suddenly increases, it is likely that the nanopore has clogged. Pause the run and tap the shielding cap firmly. Continue recording if the RMS noise is <10. If this does not reduce RMS noise, you can refer to “Strategies for pore opening” for additional strategies.
> - Particle rate should be between 200-1500 particles/min. If outside this range, prepare new sample dilutions adjusted accordingly.
> - At a minimum, you must record a sample at two separate pressures that match your calibration particle recordings for the software to calculate sample concentration.

Citation: J. Nathaniel Diehl, Amelia Ray, Lauren B. Collins, Andrew Peterson, Kyle C. Alexander, John S. Ikonomidis, Adam W. Akerman Measurement of Extracellular Vesicles with Tunable Resistive Pulse Sensing https://dx.doi.org/10.17504/protocols.io.ewov1ojnolr2/v1

This is an open access protocol distributed under the terms of the **Creative Commons Attribution License**

47 Remove pressure by pulling out pressure bar completely and then label the sample. An example label is included below:

- Investigation: Experiment name and date.
- Nanopore ID: serial number.
- Nanopore Part#: NP100.
- Sample ID: samplename#1_pressure3_dilution.1.10.
- Dilution: 10.
- Pressure: 3 (the software resets this value, include pressure setting in Sample ID).
- Electrolyte ID: 2x ME.

48 Set the pressure bar to 'Working Pressure 2' (e.g. 6 mbar). Repeat steps 40-41.

- Particle rate should increase with increased pressure. If this does not occur, the nanopore is not appropriately stabilized or the sample preparation is inadequate. The user may try higher pressures, differently diluted sample, or nanopore cleaning procedures.

49

Record 2-3 technical replicates per sample and average replicates post-hoc for more accurate concentration estimates.

- We found that this was not typically necessary as the recordings were highly similar, however it may be helpful when troubleshooting samples.
- Sample changeover procedures do not need to followed when recording technical replicates.

50 Follow "Sample changeover" procedures between samples.

Nanopore and qNano Cleaning

51 Click "Turn off" to discontinue current across the nanopore.

52 Remove pressure by pulling out pressure bar completely followed by opening the pressure valve.

Citation: J. Nathaniel Diehl, Amelia Ray, Lauren B. Collins, Andrew Peterson, Kyle C. Alexander, John S. Ikonomidis, Adam W. Akerman Measurement of Extracellular Vesicles with Tunable Resistive Pulse Sensing https://dx.doi.org/10.17504/protocols.io.ewov1ojnolr2/v1

This is an open access protocol distributed under the terms of the **Creative Commons Attribution License**

53 Remove VPM nozzle and follow the sample changeover process above.

54 Reduce the stretch to **42 mm** by turning the knob counterclockwise.

55

Remove the nanopore and rinse the nanopore from both sides with **500 µL** 70% ethanol followed by **500 µL** deionized water. Gently dry with clean with kimwipe and secure Nanopore in respective pouch and record date and usage hours on pouch.

> Cleaning the pore as described is only necessary if the nanopore will be used again. If the nanopore is at the maximum stretch time, it can be discarded in biohazard waste.

56

Rinse top fluid cell with **500 µL** 70% ethanol followed by **500 µL** deionized water and dry completely with kimwipe.

57

Remove 2x ME from bottom electrode, rinse with deionized water, and then dry with kimwipe.

58 Replace top fluid cell and shielding cap.

59 Unplug qNano until next use.

Data processing and analysis 10s

60

Citation: J. Nathaniel Diehl, Amelia Ray, Lauren B. Collins, Andrew Peterson, Kyle C. Alexander, John S. Ikonomidis, Adam W. Akerman Measurement of Extracellular Vesicles with Tunable Resistive Pulse Sensing https://dx.doi.org/10.17504/protocols.io.ewov1ojnolr2/v1

This is an open access protocol distributed under the terms of the **Creative Commons Attribution License**

Select analyze data, which will take the user to a new interface.

61

Right click on “unprocessed files” -> select “process files”.

62 Select each sample/calibration particle reading and ensure all data entries are accurate.

> The user will need to manually input the appropriate pressure for each sample at this stage.

63

Right click on sample and select “Save dataset”.

> This will save all samples/calibration particles simultaneously.

64 Ensure that each set of calibration and sample recordings have increased particle rates at higher pressures by selecting individual files in the left-most interface and then clicking the line graph icon.

65

Select the “Calibrated” tick-box next to the first sample. This will bring up a new window → select the sample recording at 2 pressures and the calibration particles at the equivalent pressure, stretch and voltage → click OK.

66

Repeat step 63 for all samples.

67

Select all samples by placing a check in all tick boxes along left most column of sample interface.

Citation: J. Nathaniel Diehl, Amelia Ray, Lauren B. Collins, Andrew Peterson, Kyle C. Alexander, John S. Ikonomidis, Adam W. Akerman Measurement of Extracellular Vesicles with Tunable Resistive Pulse Sensing https://dx.doi.org/10.17504/protocols.io.ewov1ojnolr2/v1

This is an open access protocol distributed under the terms of the **Creative Commons Attribution License**

68

Hover over blockade summary at bottom left of screen → a tab interface should appear, pin it by clicking the push pin icon in the upper right corner of the new tab interface.

69

Select the funnel icon → select "Particle Diameter (nm) → enter "<300".

70

Right click and save all datasets.

71

Apply concentration fraction right clicking on a dataset → select "Concentration Fraction" → set lower limit to **30 nm** , upper limit to **300 nm** , and check "apply to all datasets" → click "OK".

72 Repeat step 63 to recalibrate all sample sets.

73

Select dropdown menu next to "Preview" button → select "Group Report" to generate a PDF containing a simplistic report summarizing all samples and their data values.

Citation: J. Nathaniel Diehl, Amelia Ray, Lauren B. Collins, Andrew Peterson, Kyle C. Alexander, John S. Ikonomidis, Adam W. Akerman Measurement of Extracellular Vesicles with Tunable Resistive Pulse Sensing https://dx.doi.org/10.17504/protocols.io.ewov1ojnolr2/v1

This is an open access protocol distributed under the terms of the **Creative Commons Attribution License**
