# Supplementary material for: A standardized method for plasma extracellular vesicle isolation and size distribution analysis
Source: PLoS One. 2023 Apr 28;18(4):e0284875. doi: 10.1371/journal.pone.0284875 (PMC10146456; doi:10.1371/journal.pone.0284875)
Supplement: S1 Fig — Each column represents visualization of total protein loaded onto the gel (Activation), transferred onto the nitrocellulose (Transfer), or specific target detection (Immunodetection). Bio Rad TGX gels were used for protein separation by molecular weight. These gels contain a trihalo compound which modifies tryptophan residues in protein samples by a covalent modification. When exposed to ultraviolet (UV) excitation, a fluorescence signal is visualized representing total protein in both the gel (Activation) and on the nitrocellulose (Transfer). Chemiluminescent immunodetection is recorded by ChemiDoc Imaging System for each antibody exposure A) CD9 B) CD68 C) CD81 D) CD63 E) GM130 F) GAPDH G) MMP-14 H) MMP-2 I) TGFβ J) TIMP-2. Gel order: Ladder, EVs, whole cell lysate. The yellow box indicates the cropped region included in Fig 1A. (PDF) [file pone.0284875.s003.pdf]

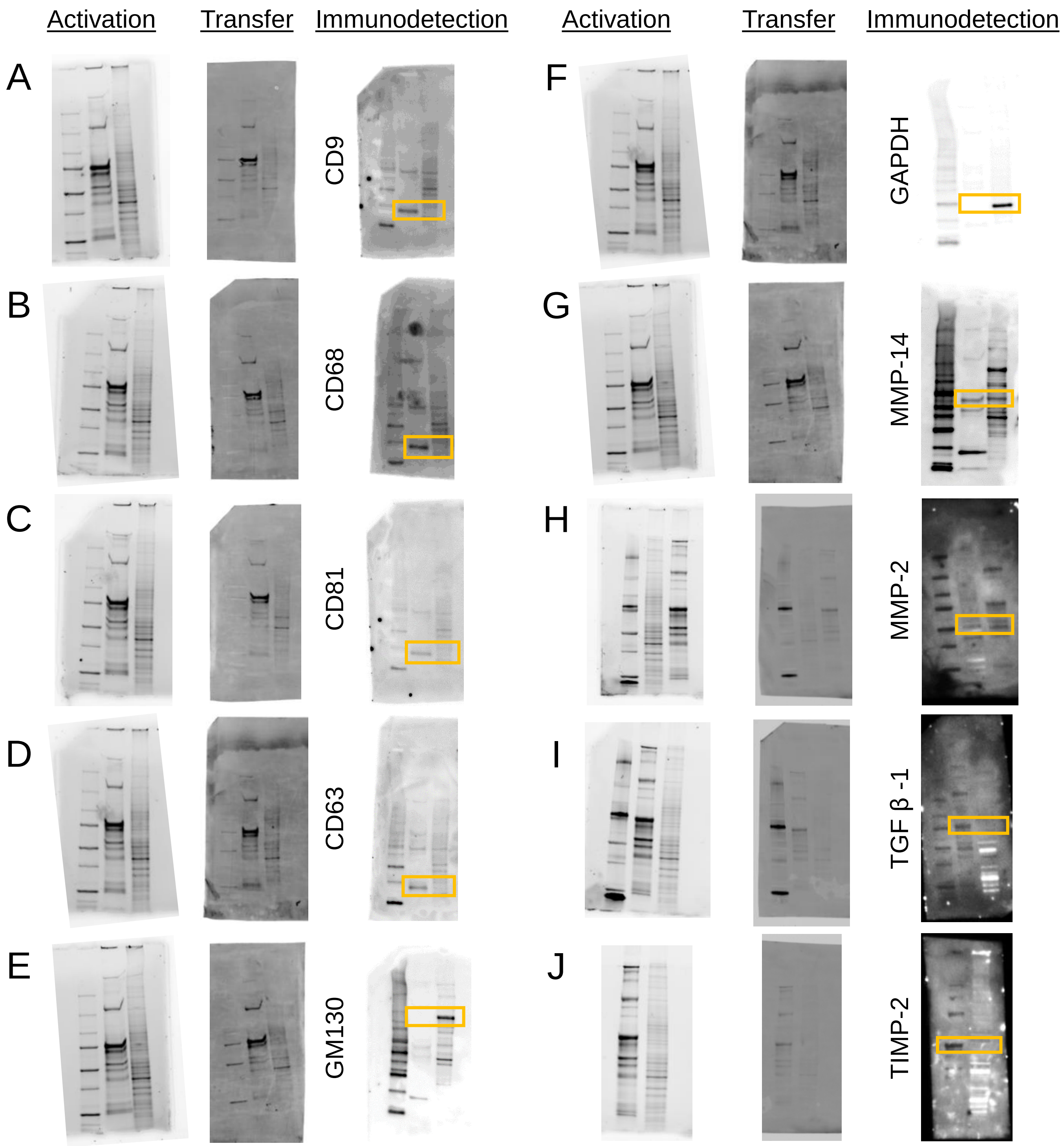

Supplemental Figure 1. Full gel/blot images accompanying figure 1A. Each column represents visualization of total protein loaded onto the gel (Activation), transferred onto the nitrocellulose (Transfer), or specific target detection (Immunodetection). Bio-Rad TGX gels were used for protein separation by molecular weight. These gels contain a trihalo compound which modifies tryptophan residues in protein samples by a covalent modification. When exposed to ultraviolet (UV) excitation, a fluorescence signal is visualized representing total protein in both the gel (Activation) and on the nitrocellulose (Transfer). Chemiluminescent immunodetection is recorded by ChemiDoc Imaging System for each antibody exposure: **A)** CD9 **B)** CD68 **C)** CD81 **D)** CD63 **E)** GM130 **F)** GAPDH **G)** MMP-14 **H)** MMP-2 **I)** TGFβ **J)** TIMP-2. Gel order: Ladder, EVs, whole cell lysate. The yellow box indicates the cropped region included in figure 1A.
